# The field phenotyping platform's next darling: Dicotyledons

**DOI:** 10.3389/fpls.2022.935748

**Published:** 2022-08-24

**Authors:** Xiuni Li, Xiangyao Xu, Menggen Chen, Mei Xu, Wenyan Wang, Chunyan Liu, Liang Yu, Weiguo Liu, Wenyu Yang

**Affiliations:** ^1^College of Agronomy, Sichuan Agricultural University, Chengdu, China; ^2^Sichuan Engineering Research Center for Crop Strip Intercropping System, Chengdu, China; ^3^Key Laboratory of Crop Ecophysiology and Farming System in Southwest, Ministry of Agriculture, Chengdu, China

**Keywords:** high-throughput phenotyping platform, dicotyledonous crops, field, research progress, development direction

## Abstract

The genetic information and functional properties of plants have been further identified with the completion of the whole-genome sequencing of numerous crop species and the rapid development of high-throughput phenotyping technologies, laying a suitable foundation for advanced precision agriculture and enhanced genetic gains. Collecting phenotypic data from dicotyledonous crops in the field has been identified as a key factor in the collection of large-scale phenotypic data of crops. On the one hand, dicotyledonous plants account for 4/5 of all angiosperm species and play a critical role in agriculture. However, their morphology is complex, and an abundance of dicot phenotypic information is available, which is critical for the analysis of high-throughput phenotypic data in the field. As a result, the focus of this paper is on the major advancements in ground-based, air-based, and space-based field phenotyping platforms over the last few decades and the research progress in the high-throughput phenotyping of dicotyledonous field crop plants in terms of morphological indicators, physiological and biochemical indicators, biotic/abiotic stress indicators, and yield indicators. Finally, the future development of dicots in the field is explored from the perspectives of identifying new unified phenotypic criteria, developing a high-performance infrastructure platform, creating a phenotypic big data knowledge map, and merging the data with those of multiomic techniques.

## Introduction

With the increasing popularity of sequencing technology and the scale of materials to be tested, a new issue has arisen: a lack of suitable high-throughput phenotype acquisition technology to obtain corresponding phenotypic information. In addition, based on a large amount of crop genome information, determining how to analyze the interaction mechanisms of gene function, plant phenotype, and environmental response efficiently and with a high resolution has become a new challenge (Furbank and Tester, 2011). In this context, genomics, corresponding to the phenomics concept, has arisen at a historic moment (Zhao et al., 2019). The essence of the phenotype is a plant genome sequence three-dimensional expression, and its regional differentiation characteristics and evolution are intergenerational (Tardieu et al., 2017; Zhao, 2019), so the plant phenotypic group contains information with a complexity far beyond the estimates. Therefore, the genotype–phenotype–environment relationship can be systematically and deeply explored from an omic perspective to reveal the response mechanism of structural and functional characteristics of plants to genetic information and environmental changes at multiple scales (Pan, 2015; Tardieu et al., 2017; Zhou et al., 2018).

Currently, crop phenotype research is primarily conducted in the United States, Germany, France, Australia, the United Kingdom, Italy, Japan, Canada, Mexico, India, and China (Xiao et al., 2021). Research objects have included maize (Souza and Yang, 2021; Xie et al., 2021; Shao et al., 2022), rice (Mishra et al., 2021; Muharam et al., 2021; Xiao et al., 2021), wheat (Prey and Schmidhalter, 2020; Furbank et al., 2021; Zelazny et al., 2021), and other monocotyledons. This focus on monocotyledons probably occurred because their morphological structure is relatively simple, and the difficulty of image acquisition and data analysis is relatively low. Leaf counting has been realized in maize, sorghum, and other monocotyledons over the entire growth period (Miao et al., 2021). However, studies on the leaves of dicotyledonous species such as soybean and cotton have focused only on comprehensive indicators such as canopy coverage and compactness due to the severe occlusion between leaves and complex plant types (Moreira et al., 2019; Li et al., 2020), which has led to the loss of many details. Leaves are closely related to plant photosynthesis, thus affecting biomass accumulation, which in turn is related to yield formation, so the loss of information is not conducive to the in-depth study of the phenotypes of dicotyledons. A significant positive correlation exists between the panicle number and the yield at maturity, and this number can be identified and counted directly at a certain regional scale in most monocotyledons. There was a significant positive correlation between panicle number and yield at the maturity stage, and it could be recognized and counted directly on a certain regional scale in most monocotyledons (Jun et al., 2021; Wanli et al., 2021). However, in many dicotyledons, researchers can predict yields using only a large number of other indicators or measure the yield by picking and laying out fruits at maturity (Casagrande et al., 2022) or by picking and spreading out fruits at maturity (Li et al., 2021; Xiaobin et al., 2022), which greatly increases labor costs and is not beneficial to the development of high-throughput phenotypes.

Branching is an important common feature of dicotyledons. The quantity of branches and their position influence yields and are connected to the lodging resistance. Studies have shown that by reducing the position of branches and increasing the number of effective branches in oilseed rape, the lodging resistance can be improved, and the yield per plant can be increased (Fan et al., 2021; Amoo et al., 2022). The branching ability guarantees the yield formation in soybean (Xiaobo et al., 2012; Yu-Shan et al., 2015). The major goal of breeders is to increase upland cotton yields by controlling the branch type and using appropriate mechanical picking methods. Therefore, Wu et al. (Wu et al., 2021; Zhan et al., 2021; Sun et al., 2022) carried out a series of studies on branching development. These studies have contributed to improved breeding by providing great genes for improving plant accessions. However, few studies involving the use of high-throughput phenotyping platforms have been conducted, which has severely slowed the breeding of dicotyledonous plants.

Dicotyledons account for 4/5 of the total number of angiosperms and play an important role in agricultural production (Chuanji, 1982). Soybean, broad bean, rape, cotton, and other dicotyledonous species are commonly cultivated and are all directly tied to human existence. According to imprecise statistics, the global demand for soybeans is ≈388 million tons per year. With an annual consumption of almost 600 million tons (searched in the U.S. Department of Agriculture data), rapeseed is the world's second-largest oil crop species. Therefore, high-throughput phenotypic studies on dicotyledons are highly important.

The current high-throughput phenotyping research environment mainly includes indoor potted plants (Bodner et al., 2021; Zea et al., 2022) because indoor imagining, which can swiftly and accurately obtain a large number of phenotype inages for later analysis and verification, faces fewer restrictive factors. Thousands of phenotypic experiments carried out in environmentally controlled growth facilities or fields each year can provide a vast amount of phenotypic data. Due to the impact of environmental variations, the replication of results by the same researcher and the repeatability of results in separate tests by other laboratories are frequently unsatisfactory (Poorter et al., 2012). Environmental aspects are critical and should be given at least as much attention as the characteristics being assessed, which leads to the next question: how does one quantify all environmental impacts? The phenotyping platform is systematically presented in this paper, and the determination of the phenotype of dicotyledons against the backdrop of the rapid development of the field phenotyping platform is discussed. This paper guides investigating high-throughput phenotypic application technology for dicotyledons in the field, enhancing precision agriculture and increasing genetic gains.

## Overview of high-throughput phenotyping of dicotyledonous crops in the field

We collected statistics on field phenotyping facilities and the number of publications since 2010 to better understand how the high-throughput phenotyping of field dicot crops should be developed. In 2016,according to statistics from the International Plant Phenotype Network (IPPN), the phenotyping platform is used in the United States, Australia, China, Germany, and other countries. Nearly 200 large-scale phenotyping facilities are in operation around the world (the most notable being the Australian National Plant Phenotyping Facility “Plant Accelerator,” the British National Plant Phenotyping Center, the German Julich Phenotyping Research Center, the Netherlands Plant Eco-phenotyping Centre, and the German IPK Greenhouse Automation). There are ≈82 indoor mechanized phenotyping platforms and 81 European field mechanized phenotyping platforms (including 26 intensive and 55 barren types). Asian field mechanized phenotyping platforms are yet to be counted. However, over the last 5 years, many countries, led by the United States and China, have increased their investments in field mechanization platforms.

**Figure 1:**
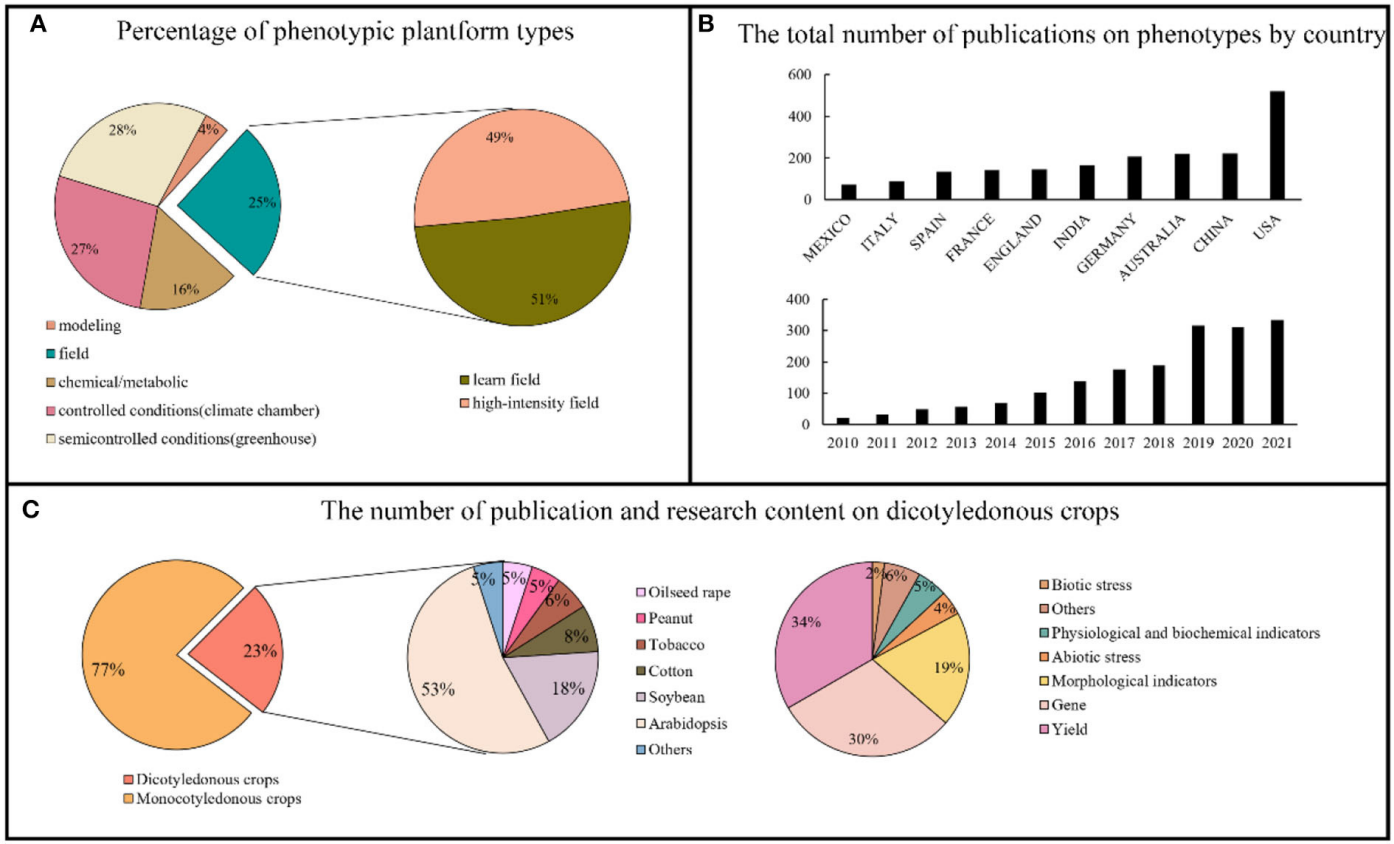
Overview of high-throughput phenotyping research. **(A)** The proportion of different phenotypic platforms and the composition of phenotypic platforms in the field (searched in IPPN, https://www.plant-phenotyping.org/ippn-survey_2016). **(B)** Ranking and annual number of phenotypic papers published by countries in the world (2010–2021) (searched in Web of Science). **(C)** Proportion of high-throughput phenotypes studied in monocotyledons and dicotyledons and the main subjects studied in dicotyledons (searched in Web of Science).

The development of phenotyping platforms has provided a solid foundation for crop phenotyping research. Only 25% of all global high-throughput phenotyping platforms are used for field research, and only 49% of the platforms are actually used to obtain high-throughput phenotyping information in the field (Figure 1A). The number of papers published on crop phenotypes has increased annually; in 2019, the number of papers published each year had surpassed 300. Additionally, among many countries and regions that are involved in high-throughput phenotyping studies of plants, the United States is ahead in terms of research results. We list only the top 10 countries or regions in Figure 1B. Dicotyledonous crops account for only 23% of the research results, which is substantially lower than monocotyledons. Arabidopsis is the most studied dicot crop, most likely because it is a commonly used model crop and using it to analyze new phenotypes can greatly decrease the difficulty of research. Furthermore, soybeans, cotton, tobacco, peanuts, rape, and other crops have gradually entered the academic research field. Finally, we compiled statistics on dicotyledonous crop research topics. According to the statistical findings, dicotyledonous plant research topics mainly focus on six aspects: yields, physiology and biochemistry, genes, biotic stress, abiotic stress, and growth dynamics. Genes were the most studied topic, followed by yield (Figure 1C). These research topics are covered in the following chapter.

## Research progress of field high-throughput phenotypic information platforms

**Figure 2:**
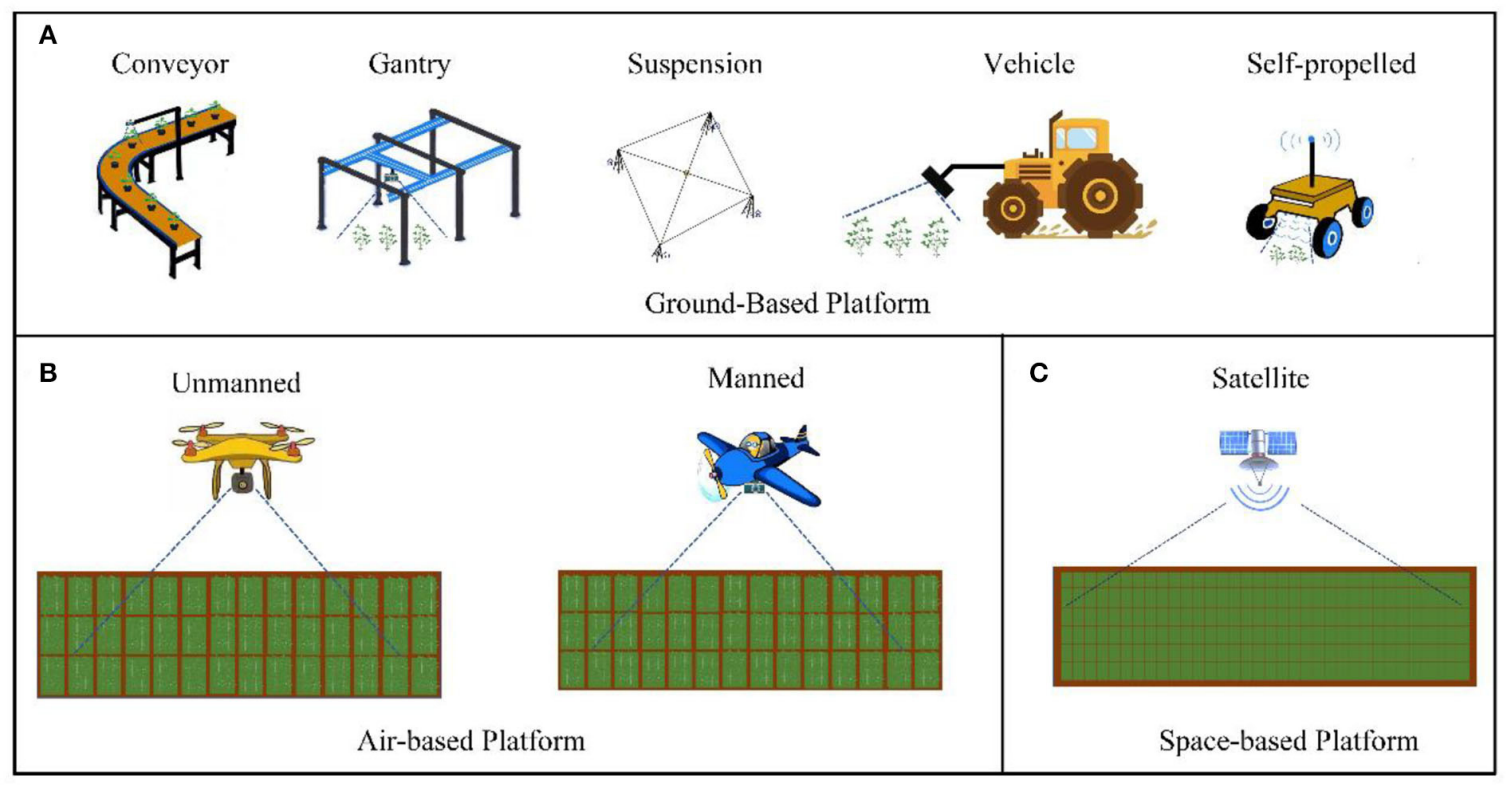
Field high-throughput phenotypic information platform. **(A)** Ground-based platforms include conveyor belt types, gantry types, suspension cable types, vehicle types, and self-propelled types. **(B)** Air-based platforms include UAVs or manned aircraft. **(C)** Space-based platforms include satellite remote sensing.

Platforms are generally classified into three types based on the different spatial areas in which they operate: ground-based platforms, air-based platforms, and space-based platforms (Huichun et al., 2020). Ground-based platforms encompass all plant phenotyping platforms that are in contact with the ground while being built or used. Based on their loading modes, sensors are classified into conveyor belt types, gantry types, suspension cable types, vehicle types, and self-propelled plant phenotyping platforms (Figure 2A). Air-based platforms include all platforms that collect phenotypic data in the air, which are classified as unmanned aerial vehicles (UAVs) or manned aircraft depending on whether or not a human pilot is present (Figure 2B). Space-based platforms collect phenotypic data using satellite remote sensing (Figure 2C).

### Ground-based platforms

Research has been conducted on ground-based platforms, with Crop Design in Belgium being the first company in the world to develop a commercialized large-scale phenotyping measurement platform (Reuzeau et al., 2006). Foundation platforms have advantages and disadvantages in their use (Table 1). The conveyor belt-type foundation platform can detect plant phenotypic indicators in real time, and the detection objects can be flexibly replaced based on individual needs. However, this device is better suited for indoor use. The gantry-type platform has a walking device, an automatic control module for mechanical motion, and a high-precision sensor array. It is not affected by the environment, has a low impact, and can take measurements repeatedly every day. However, the cost is high, and only a fixed area can be observed. Suspension-type platforms have the advantages of not requiring guards, continuous operation (such as at night), good repeatability, and high measurement accuracy, but they are typically expensive and can detect only a limited number of areas. Vehicle-mounted platforms are typically agricultural machinery platforms, such as tractors, that are outfitted with various sensors to form phenotype platforms. They can meet the application requirements of most researchers and small businesses to the greatest extent possible due to their low cost, constant perspective, easy installation, and simple operation. However, due to the wide wheel, low body height, and high vibration, agricultural machinery is primarily suitable for short plants and is limited by row spacing and plant space. Currently, the vehicle-borne phenotypic platform is being used to collect biomass (Busemeyer et al., 2013), plant height (Comar et al., 2012), leaf area, stem diameter, canopy temperature, and other phenotypic data for wheat (Andrade-Sanchez et al., 2013), cotton (Sun et al., 2018), and other crops. Researchers should examine the impact of the weight of agricultural machinery on the soil structure and the root system of plants. An increasing number of researchers are experimenting with compact self-propelled ground mobile platforms to carry phenotyping sensors to minimize costs, increase the measurement accuracy, and reduce environmental side effects (Bai et al., 2016; Young et al., 2018). Nonetheless, business solutions are lacking, making promotion extremely difficult. Furthermore, by using the chassis of commercial self-propelled ground rovers, several researchers have created phenotypic platforms for various applications (Shafiekhani et al., 2017), offering a novel approach to the creation of self-propelled phenotyping platforms.

**Table 1:** Performance comparison of plant phenotypic information collection platforms.

**Phenotypic platform**	**Platform name**	**Research and development unit**	**Platform type**	**Advantages**	**Limitations**	**Scanning scale**	**Practical application**
Ground-based Platform	Crop Observer	PhenoVation, Netherlands company	Conveyor belt	Real-time measurement of photosynthetic efficiency, estimation of soil coverage by plant leaves	More suitable for indoor work	1–10 m^2^	Experiments in a test field at Wageningen University in the Netherlands
	Field Scan	PhenoVation, Pheno Spex company	Gantry type	Not affected by the environment, the efficiency can reach 5,000 plants/h, and the measurement can be repeated every day	High cost, only a fixed area can be observed	10–50 m^2^	Applied to the field phenotyping platform built by Nanjing Agricultural University in 2018
	Field Scanalyzer	Germany, Lemna Tec company	Gantry type	With walking device, automatic control module of mechanical movement, high-precision sensor array, supporting data acquisition and analysis software	High cost, only a fixed area can be observed		Procurement by scientific research institutions such as French Academy of Agricultural Sciences, Chinese Academy of Sciences and DuPont Pioneer (Virlet et al., 2016)
	Breed Vision	University of Applied Technology, Osnabruck, Germany	Gantry type	Mobile darkroom (moving speed 0.5 m/s), equipped with 3D depth camera, color camera, laser ranging sensor, light screen imaging Settings and other optical equipment	High cost, only a fixed area can be observed	1–10 m^2^	University of Applied Technology, Osnabruck (Busemeyer et al., 2013)
	Spidercam	University of Nebraska-Lincoln	Suspended cable	Covering a field of 4,000 m^2^, a variety of sensors can be mounted on the suspension cable platform	High cost, only a fixed area can be observed	50–100 m^2^	Test field use at the University of Nebraska-Lincoln in 2017 (Ge et al., 2019)
	ETH	Swiss ETH Field Phenotyping Platform	Suspended cable	Suspended various sensors	High cost, only a fixed area can be observed	100–1,000 m^2^	ETH plant research station Lindau-Eschikon (Kirchgessner et al., 2016)
	Field Scanalyzer	UK, Rothamsted Research Centre	Suspended cable	Equipped with a variety of sensors, the applicability is strong, the system runs smoothly, and is less affected by external interference	High investment cost, high operation and maintenance costs, not suitable for large breeding areas	50–100 m^2^	/
	Phenotyping Robot	USA, Iowa State University	Self-propelled	Multiple stereo cameras trigger synchronously, and multiple sets of stereo lenses are superimposed to ensure phenotypic analysis of tall crops	No commercial solution, need to design independently	1–10 m^2^	Used in the experimental field of Iowa State University in 2014
	GPheno Vision	University of Georgia	Vehicle	Low cost, can be equipped with a variety of sensors	Fuel power, larger vibration, wider tires, and requirements for row spacing		In 2017, it was used in the experimental field of the University of Georgia, USA (Jiang et al., 2018)
Air-based Platform	Helipod	CSIRO	UAV	Equipped with thermal imager and RGB camera to obtain canopy temperature and RGB images	Limited load capacity, regulated altitude, short flight time, and affected by the environment	100–2,000 m^2^	Intensive phenotyping experiments in Canberra, Australia
	LiAir	Beijing Digital Green Earth Technology Co., Ltd.		Wide field of vision, daily measurement 2 km^2^			In 2012, it has been applied to the field of agroforestry phenotyping
Space-based Platform	The Pleiades−1A and Worldview-3	/	Satellite	The detection area is the largest, which is convenient for macro-control	Highest cost, relatively low accuracy, only suitable for large area inspection	>10,000 m^2^	Disease and Crop Water Stress Detection

ETH, Ethiopia; UK, Britain; CSIRO, Commonwealth Scientific and Industrial Research Organisation; UAV, unmanned aerial vehicle.

The short line (-) indicates that the platform is not mentioned in the article.

Li et al. (2020) identified new I-trait indicators (plant density, relative frequencies, and entropy, among others) that accurately reflect the response of cotton to drought stress at the seedling stage. By merging high-throughput phenome, genome, and transcriptome data, researchers found that two unannotated genes, GhA040377 and GhA040378, were considerably upregulated in response to drought. Finally, this study advocated the use of phenomics to improve the genetics of cotton and was the first phenomics research publication on drought resilience in cotton.

### Air-based platforms

Air-based platform research is still in its early stages, but it is progressing quickly. This type of platform has the advantage of scanning a large area of land in a short period of time, but there are also some drawbacks such as a low information accuracy, an insufficient payload, limited endurance, and weather vulnerability. Currently, air-based phenotyping platforms primarily include UAVs and manned helicopters. When compared to manned helicopters, UAVs have lower costs, lower flying altitudes, and superior information acquisition precision. As a result, numerous studies have been conducted on the acquisition of field phenotypic information *via* UAVs. The number of sensors that a UAV can carry is limited due to its low payload capacity compared to ground-based platforms. Remote sensing analysis of crop phenotypes (Liu et al., 2016) and maturity evaluation (Malambo et al., 2018) is performed using RGB cameras, infrared imaging, multispectral/hyperspectral cameras, and other sensors. Disease diagnosis (Sugiura et al., 2016), yield estimation (Chang et al., 2021), growth state monitoring and evaluation (Jin et al., 2017; Hu et al., 2018), and analysis of critical phenotypic features have been performed using this type of platform (Ding et al., 2019). Trevisan et al. (2020) used a 3D point cloud derived from UAV images to develop a method for detecting sorghum spikes. The correlation coefficients between the average panicle length and width assessed by UAVs and those measured on the ground were 0.61 and 0.83, respectively. Karthikeyan et al. (2020) used a space-based platform to collect images twice a week and then employed two complimentary convolutional neural networks (CNNs) to forecast soybean maturity. This method can detect the sources of mistakes in maturity forecasting, and its architecture overcomes earlier research limitations and can be used in large-scale commercial breeding initiatives.

When UAVs are employed, it is also important to consider its stability, safety, and controllability.

### Space-based platforms

Satellite platforms for monitoring the status of large areas of crops are referred to as a “space-based platforms.” Because they have the highest data flux and lowest accuracy, space-based phenotyping platforms are suited only for a wide spectrum of detection. On the one hand, satellites have a massive payload, and onboard sensors can cover optical, thermal, microwave, and fluorescence frequencies, allowing for the collection of large amounts of data in a short period of time. On the other hand, satellites can provide recurrent information on agricultural conditions at different scales throughout the season (including yield forecasting, field preparation, crop health monitoring, irrigation, and site-specific management) (Zhang et al., 2020). Furthermore, improvements in the spatial (Weiss et al., 2020), spectral, and temporal resolutions of satellite measurements have increased their use in plant breeding (Prey et al., 2020; Weiss et al., 2020). Space-based platforms have become increasingly popular in recent years, and despite their high cost, they are being used in a limited number of agricultural applications. For example, NASA has created the Space Test Station for Thermal Radiation of Ecological Systems, which can be used to monitor the soil moisture content (Entekhabi et al., 2010), drought warnings, and water usage efficiency (Reynolds et al., 2019b). Soil Moisture Active Passive (SMAP) observations of soil moisture and freeze/thaw timing can reduce a major uncertainty in quantifying the global carbon balance by helping to resolve an apparent missing carbon sink on land over the boreal latitudes (Entekhabi et al., 2010). Pleiades-1a and World View-3 have been utilized to detect disease and agricultural water stress (Navrozidis et al., 2018; Salgadoe et al., 2018), promoting the advancement of precision agriculture. On the practical side, Jain and Balwinder-Singh (2019) demonstrated how microsatellite data management can have a substantial impact on agricultural sustainability in underdeveloped nations.

In summary, many crop phenotyping platforms in the field have distinct properties. Ground-based platforms can analyze the largest number of species and are well-established. Their price is reasonable; however, the vision height is limited, and the data throughput is modest. Two types of space-based platforms are available: manned and unmanned. Unmanned platforms can quickly acquire macrolevel information in a specific area, with broad vision and enormous data throughput, but their cost is significant. Space-based platforms, which offer the widest observation area, mostly rely on satellite remote sensing. However, they cannot perform small-scale or refined crop detection and are infrequently employed in agriculture due to their high cost. The use of space-based platforms in agriculture is projected to become more common as science and technology advance. The properties of many types of field phenotyping platforms are shown in Table 1. When employing phenotyping platforms, platforms that are reasonable for the circumstances must be chosen and developed, taking into account actual requirements such as mobility, ease of operation, data flux and accuracy, and costs (Lee et al., 2015).

## Research status of high-throughput phenotypic information of dicots in the field

### Morphological indicators

Plant morphology has essential biological implications in agricultural production, and it is a key component of plant science research. Traditional plant identification and classification approaches rely on professional experience, which is subjective and inaccurate, to examine plant morphological traits such as appearance, shape, texture, and color (Liu et al., 2016). Using machine vision, picture segmentation, and big data processing technologies to reliably gather and analyze crucial plant traits is an important technical means for the development of contemporary agriculture, with significant guiding value for crop management and genetic breeding (Granier and Vile, 2014; Li et al., 2021). Scholars have conducted field studies on the morphological indicators of dicotyledonous crops, including stem height (Paproki et al., 2012), plant height (Sun et al., 2017), leaf width (Paproki et al., 2012), leaf length (Paproki et al., 2012), number of leaves (Dobrescu et al., 2020), canopy coverage (Kirchgessner et al., 2016; Borra-Serrano et al., 2020; Wan et al., 2021; Xu et al., 2021), canopy height (Kirchgessner et al., 2016; Borra-Serrano et al., 2020), canopy roughness (Herrero-Huerta et al., 2020), and flowers (Xu et al., 2017; Jiang et al., 2020).

Stems and leaves are the most frequently utilized factors for crop morphological indication, and RGB values are commonly used by researchers to extract these parameters. For example, Paproki et al. (2012) created a cotton plant model by capturing RGB images of cotton plants and extracting indicators such as the cotton stem height, leaf width, and leaf length. When compared to manual measurements, the average absolute error was 9.34, 5.75, and 8.78%, respectively, while the correlation coefficients were 0.88, 0.96, and 0.95, respectively. Dobrescu et al. (2020) used deep learning to analyze the number of Arabidopsis leaves in RGB photos, and the *R*^2^ = 0.92 when compared to manual measurements. The approach of collecting crop morphological data from RGB images is quite accurate.

The extraction of crop canopy information is useful in the study of crop data. Previous research has demonstrated that thermal imaging, multispectral imaging, and RGB imaging can be utilized to monitor soybean canopy coverage and canopy height with an accuracy of >90% (Sun et al., 2017; Borra-Serrano et al., 2020), and an *R*^2^ > 0.5 was found after linear fitting using the values measured in the field (Herrero-Huerta et al., 2020). Moreover, similar research has been conducted on cotton, rape, and other crops. Xu et al. (2021) created a UAV system with three cameras (RGB, multispectral, and thermal) and a lidar sensor to identify cotton canopy coverage and canopy height, with an average relative error of only 6.6%. The approach of collecting crop morphological data from RGB images is quite accurate. Using a UAV platform fitted with an RGB and multispectral camera, Wan et al. (2021) obtained rape canopy images. The PROSAIL-GP model was used to invert rapeseed vegetation coverage and the *R*^2^ = 0.79. The resilience of the proposed method was confirmed in cotton (*Gossypium hirsutum* L.), and a better retrieval accuracy was obtained.

Many experiments have been conducted to analyze cotton flowering utilizing high-throughput phenotyping approaches. Color RGB images obtained by a UAV system and a CNN were used to detect the number of cotton blossoms in the original image, with an error of just −4~3 (Xu et al., 2017). Scanning cotton with a tractor-mounted lidar had an *R*^2^ = 0.98 compared with that of manual measurements (Sun et al., 2017). Moreover, cotton flowering status can be recognized using multiview color imaging and deep learning, with an *R*^2^ = 0.88 and an RMSE = 0.79 (Jiang et al., 2020).

Previous research has indicated that the use of a high-throughput phenotyping platform to obtain crop morphological indicators is nearing maturity. Researchers are more likely to use a ground-based platform equipped with an RGB camera as the primary research tool in the study of crop leaves and stalks. Canopy information can be extracted using UAVs equipped with RGB, hyperspectral, and radar sensors, but the accuracy is slightly lower than that obtained in leaf and stem studies. In terms of flower counting, UAVs and vehicle-mounted platforms outfitted with RGB cameras and lidar are commonly used, and the accuracy is acceptable. Thus, it is not surprising that the choice of a high-throughput phenotyping platform is closely related to the specific indicators being studied.

### Physiological and biochemical indicators

Crop physiological and biochemical indices include chlorophyll, photosynthetic rate, water stress, biomass, salt tolerance, and leaf water content. These indices can accurately reflect crop growth, health, and resistance. Crop physiology and biochemistry studies involving the use of a high-throughput phenotyping platform have primarily focused on the leaf color (Bai et al., 2018), element content (Naik et al., 2017; Prananto et al., 2021), biomass (Yoosefzadeh-Najafabadi et al., 2021), and water use efficiency (Thorp et al., 2018).

To score iron deficiency chlorosis, RGB images of soybean plants in the field were collected, which revealed an overall accuracy of >81% (Naik et al., 2017; Bai et al., 2018). Prananto et al. (2021) used a ground-based platform equipped with a near-infrared spectrometer (wavelength range 1,350–2,500 nm) to estimate different macro- and micro-elements in cotton leaf tissues, with accuracies of 87.3 and 86.6%, respectively. The fitting degree of aboveground fresh biomass can be as high as 0.91 (Yoosefzadeh-Najafabadi et al., 2021) when combining hyperspectral photography with deep neural network (DNN) analysis for biomass acquisition and water use efficiency. Multispectral images can be used to determine the crop canopy coverage, which is then used to estimate the coefficient of basic crops to improve the crop water use efficiency (Thorp et al., 2018). Previous studies have shown that by acquiring high-throughput phenotypes, crop nutrients can be estimated in the field, farmers can proactively manage nutrition to avoid yield losses or environmental impacts, and evidence is provided for crop selection.

In short, physiological and biochemical indices have received less attention than morphological indices have. The platforms are mainly based on the ground and in space, and the sensor types are more complicated and varied. This research will help with nutrient decisions and the breeding of new varieties.

### Biotic/abiotic stress indicators

Pests and diseases are the primary causes of crop yield reductions in terms of biological stress. Abiotic stress refers to all the factors that negatively affect crop growth and development as a result of an unsuitable external environment, which mainly includes light, temperature, water, and fertilizer. Crops are increasingly subjected to biotic/abiotic stress during growth as the global climate changes. The goal of smart agricultural plant protection is to locate the type of stress and determine the degree of stress through accurate identification before crops are stressed and irreparable damage is caused to protect plant operations.

The traditional method for evaluating the tolerance of crops to external stress in terms of the field conditions is to judge the damage level visually, but this method is labor intensive and susceptible to subjective error. This problem can be effectively resolved by utilizing high-throughput phenotyping. He et al. (2019) used RGB images of rapeseed to judge rapeseed insect pests with an accuracy of 77.14% using CNN processing and analysis. Naik et al. (2017) and Ghosal et al. (2018) used high-throughput phenotypes to evaluate soybean under abiotic stress and obtained promising results. Soybean field images were captured using a UAV equipped with a multispectral and infrared thermal imager, and five image features were extracted, including the canopy temperature, normalized difference vegetation index, canopy area, canopy width, and canopy length. The damage level was evaluated by a deep learning model, with an accuracy of 0.9 based on these features. The method proposed in this paper appears to be very promising for soybean breeding, and it is expected to replace an abundance of manual operations and more efficiently assess the level of waterlogging disasters (Zhou et al., 2021). High-throughput phenotypes have enormous potential for measuring crop traits and detecting crop responses to biological or nonbiological stresses.

### Yield indicators

Crop yield estimation in the field is regarded as the foundation of food security. In recent years, remote sensing information and crop growth models have been coupled to resolve a variety of agricultural problems, such as crop growth detection and yield prediction.

Bai et al. (2016) collected soybean field traits *via* a self-propelled platform outfitted with five sensor modules (ultrasonic distance sensor, thermal infrared radiometer, normalized difference vegetation index (NDVI) sensor, portable spectrometer, and RGB network camera). The results of the analysis and processing revealed that the traits obtained by the sensors were highly correlated with the final grain yield in both the early and late seasons (*r* = 0.41–0.55, and *r* = 0.55–0.70). For example, Moreira et al. (2019) attached an RGB camera to a UAV to collect soybean production and canopy coverage data and continued the analysis to yield | ACC with an actual output correlation of 0.75. Yoosefzadeh-Najafabadi et al. (2021) used the hyperspectral vegetation index (HVI) collected by a UAV equipped with hyperspectral sensors to predict soybean yields in conjunction with two artificial intelligence algorithms integrated baggies (EB) and DNN and obtained determination coefficients (*R*^2^) of 0.76 and 0.77, respectively.

We suggest that to acquire a yield index, we must first acquire multi-index information. Because different researchers utilize different predictors, different high-throughput phenotypic platforms and sensors can be used. In general, the use of UAVs equipped with RGB cameras is the most common method. Yield prediction is beneficial for shortening the breeding time of varieties, reducing the cost of yield measurements, and enhancing the yield measurement efficiency, all of which are vital in crop research and development. Table 2 provides statistics on the field phenotypic information of dicotyledons.

**Table 2:** Statistics of field phenotype research on dicotyledonous crops.

**Classification of indicators**	**Crop category**	**Type of data**	**Phenotypic analysis method**	**Phenotypic parameters**	**Accuracy %**	***R*** **^2^**	**Shooting scale**	**Year**	**Author**
**Morphological indicators**	Cotton	RGB	3D reconstruction	Stem height, leaf width, leaf length	91.66, 94.25, 91.22	–	Single	2012	Paproki et al., 2012
	Soybean	Thermal, Multispectral	Machine learning	Canopy coverage, canopy height	–	0.86, 0.99	Single	2016	Kirchgessner et al., 2016
	Cotton	RGB	CNN	Number of flowers	Error = −4~3	–	Single	2017	Xu et al., 2017
	Rapeseed	Multispectral, RGB	Machine learning	Canopy coverage	–	0.79	Group	2021	Wan et al., 2021
	Soybean	RGB	Machine learning	Canopy coverage, canopy height	90.4, 99.4	–	Group	2020	Borra-Serrano et al., 2020
	Cotton	RGB	CNN	Flowering patterns	–	0.88	Single		Jiang et al., 2020
	Soybean	RGB	SFM	Canopy roughness	–	>0.5	Group		Herrero-Huerta et al., 2020
	Cotton	RGB	Metashape, Python	Canopy coverage	93.4	–	Group	2021	Xu et al., 2021
	Arabidopsis	RGB	CNN	Number of leaves	–	0.92		2020	Dobrescu et al., 2020
	Cotton	Lidar	3D point cloud	Plant height	–	1	Single	2017	Sun et al., 2017
**Physiological and biochemical indicators**	Soybean	RGB	Machine learning	Leaf iron deficiency chlorosis	>81, 96	–	Regional	2018	Bai et al., 2018
								2017	Naik et al., 2017
	Cotton	Near Infrared Spectroscopy	/	Leaf macro and micronutrients	87.3, 86.6	–	Organ	2021	Prananto et al., 2021
	Soybean	Hyperspectral	DNN	Fresh biomass of above ground	–	0.91	Group	2021	Yoosefzadeh-Najafabadi et al., 2021
	Cotton	Hyperspectral	/	Coverage, water use efficiency	–	–	Group	2018	Thorp et al., 2018
	Soybean	Spectral Scanner	Modeling	εe, εc	–	0.68	Organ	2021	Keller et al., 2021
**Biotic/Abiotic Stress**	Rapeseed	RGB	CNN	Oilseed rape pests	77.14		Regional	2019	He et al., 2019
	Rapeseed	RGB	Machine learning	Fruiting bodies of *Leptosphaeria maculans*	–	0.87	Regional	2019	Bousset et al., 2019
	Soybean	RGB	DCNN	Nonbiological	–	–	Regional	2018	Ghosal et al., 2018
	Soybean	RGB	Machine learning	Leaf iron deficiency chlorosis	96%	–	Single	2018	Naik et al., 2017
	Soybean	Multispectral, Infrared	Machine learning	Flood	–	0.9	Organ	2021	Zhou et al., 2021
**Yield**	Soybean	RGB	/	Canopy coverage	–	0.4–0.7	Regional	2016	Bai et al., 2016
	Soybean	RGB	Machine learning	Yield and maturity	–	0.51, 0.82	Group	2020	Borra-Serrano et al., 2020
	Soybean	RGB	/	Yield/canopy cover	–	0.75	Group	2019	Moreira et al., 2019
	Soybean	Hyperspectral	DNN (EB)	Yield	–	0.76, 0.77	Group	2021	Yoosefzadeh-Najafabadi et al., 2021

RGB, an abbreviation for the three primary colors; CNN, convolutional neural network; DCNN, deep convolutional neural network; SFM, multiview structure from motion; EB, integrated baggies εe, photochemical energy; εc, biomass.

The slash (/) indicates the ratio.

The short line (–) indicates that it is not mentioned in the article.

### High-throughput phenotyping and genetic breeding of dicotyledonous crops in the field

Crop breeding has gone through three generations, with the first being artificial screening as the primary method, the second being hybridization as the primary method, and the third being molecular markers and genome-wide association analysis as the primary and auxiliary methods. The fourth generation of intelligent-assisted breeding with big data, supported by multidisciplinary and multiomics data, is currently underway (Wallace et al., 2018). The incorporation of phenotypic and genomic data, as well as proteome, transcriptome, metabolome, and other omics data, is required for the fourth generation. Utilizing genome-wide association studies (GWASs), quantitative trait loci (QTL) analysis, and other technical methods, a large number of candidate genes and candidate molecular markers have been identified. Models, such as breeding information simulation, parental selection recommendation, breeding path recommendation, and breeding variety prediction, have been established to form the ultimate intelligent breeding decision system (Wang and Xu, 2019).

The high-throughput phenotyping platform, which enables the accurate assessment of a large number of field plots with a variety of measures in a short period of time, simplifies the routine quantification of crop development, physiology, and phenological characteristics (White et al., 2012; Araus and Cairns, 2014). These data provide a useful framework for addressing phenotypic bottlenecks in plant breeding (Furbank and Tester, 2011; Araus and Cairns, 2014; Kumar et al., 2015). Crop dwarfing has contributed to the growth of yields in the Green Revolution (Hammer et al., 2009; Swaminathan, 2014), and the fourth generation of intelligent-assisted breeding with big data could be the next breakthrough in accelerating the genetic harvest of crops in the field. Heritability and genetic gain potential will improve with high-throughput and precise phenotypes (Araus et al., 2018). Numerous successful results have been obtained in many crops by incorporating genomic and phenotypic data. The function of a large number of unknown genes, for example, has been quickly decoded, thereby improving the understanding of G-P maps (Raman, 2017). The water conditions of soybeans with different genotypes have been isolated (Braga et al., 2020), mutant and wild types have been effectively classified (Dobrescu et al., 2020), and flowering patterns in plants with complex canopy structures (such as cotton) have been identified (Jiang et al., 2020). Phenotypic indicators with strongly inherited traits are being investigated. Furthermore, phenotypic and genetic variation can be interpreted using optical sensing-based phenotyping (OSP) data analysis (Xavier et al., 2017; Sun et al., 2021).

### High-throughput phenotyping and precise management of dicotyledonous crops in the field

Because agriculture is currently facing resource shortages and serious farmland environmental pollution, the implementation of precision agriculture demonstrations and research is critical. High-throughput phenotyping platforms can be used to obtain crop image traits and conduct modeling to estimate the yield and quality of a crop (Xu et al., 2021) and to analyze the relationship between crop growth and environmental factors, thereby enabling more precise management (Maimaitijiang et al., 2019).

In particular, high-throughput phenotypic data can be used to analyze the factors influencing yield differences among plots, treat different plots differently, and implement “prescription farming” based on positions, regulations, and needs. Making full use of information acquisition means analyzing the crop nutrition status and the spatial and temporal changes in pests and diseases to make tillage and field management decisions, as well as investing in agricultural resources such as water and fertilizer based on local conditions. This approach can ensure that the crop production potential is fully realized and avoid the serious consequences of the overuse of chemical fertilizers and pesticides, such as increased production costs, the pollution of farmland soil and water environments, and a decline in the quality of agricultural products. To achieve the best effect and the lowest cost, agricultural outputs can be increased, the quality can be enhanced, costs can be decreased, resources can be conserved, pollution can be reduced, and the environment can be protected. As a result, precision agriculture can produce significant economic and ecological benefits, which are vital for maximizing the production potential of cultivated land, efficiently using agricultural production factors, and preserving the farmland environment.

## Development direction of high-throughput phenotypic information research on dicotyledonous field crops

**Figure 3:**
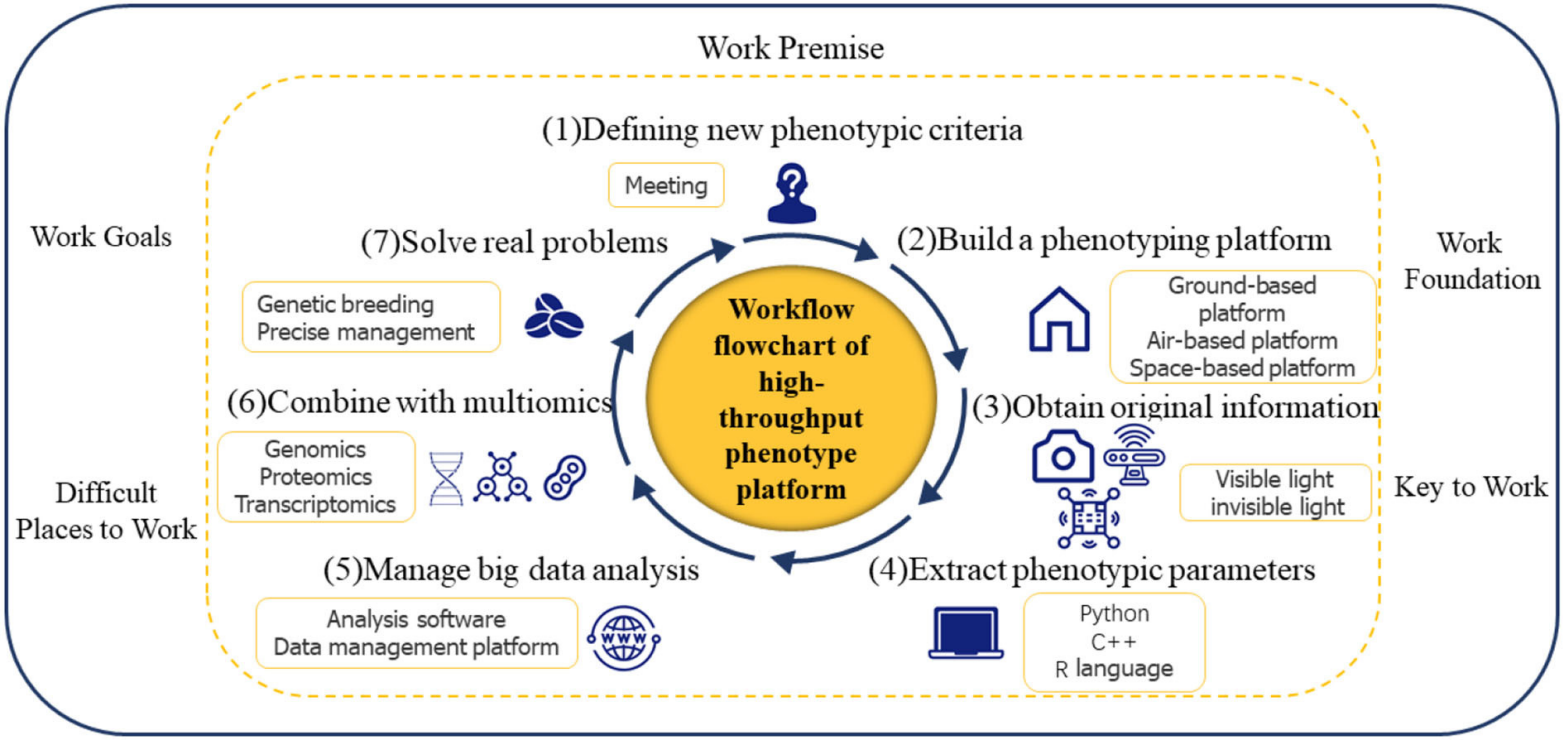
High-throughput phenotype workflow flowchart.

The development of a high-throughput phenotype must follow a certain workflow. As shown in Figure 3, from the determination of phenotypic concepts to the establishment of phenotypic platforms, the acquisition of original information, the extraction of phenotypic parameters, the analysis, processing and mining of big data, and joint analysis with multiple omics, practical problems can finally be solved. In fact, industry experts must decide on new phenotypic criteria because the step of “determination of unified new phenotypic criteria” is a premise for high-throughput phenotypic work; the demands of researchers for high-quality data, particularly in terms of resolution and accuracy, vary depending on the distinct objective features they are pursuing. Therefore, the key to collecting original information is to “develop a high-performance infrastructure platform,” but doing so is difficult. For better trait selection in breeding programs, more effective field management in agricultural operations, and the eventual augmentation of the germplasm of grain, the “construction of a knowledge map of phenotypic big data” stage is crucial but also difficult. The process of “combining with multiomics” is critical for finding functional genes and speeding up breeding.

### Determination of new unified phenotypic criteria

Healthy, sustainable development must be based on consistent standards. Mendel, the father of genetics, began to define and evaluate phenotypes as early as 1866. He described seven pairs of relative characteristics of peas in his famous paper “Plant Hybridization Experiments,” including round and wrinkled seeds, tall stems vs. short stems, green pods vs. yellow pods, and so on. In 1911, Wilhelm Johannsen, a Danish geneticist, established the concept of a biological phenotype, claiming that an organism's phenotype was the consequence of a complex interaction between the genotype and environmental circumstances (Johannsen, 1911). In the 1990s, Nicholas Schoork, an epidemiology and biostatistics expert at Case Western Reserve University, was the first to propose the concept of physics as a counterpart to genomics (Zhao et al., 2019). Since then, studies on single phenotypes or series of phenotypes of humans, animals, and plants have piqued public interest (Siebner et al., 2009), and such studies have gradually evolved into an important branch of biology (Bilder et al., 2009; Houle et al., 2010; Tester and Langridge, 2010). Plant phenotyping research began at the end of the 20th century with the goals of obtaining high-quality and repeatable shape data and quantitatively analyzing the interactions between genotypes and environmental types and the effects on yields, quality, stress tolerance, and other related main traits (Ribaut et al., 2010). Fiorani and Schurr (2013) proposed a new definition of the plant phenotype in 2013, describing it as a collection of methods and protocols for accurately measuring the growth, structure, and composition of plants at various scales. In Zhao (2019) updated the definition of a phenotype, stating that it is a physical, physiological, and biochemical mechanism that can reflect the structural and functional characteristics of plant cells, tissues, organs, plants, and populations. In essence, a phenotype is the three-dimensional sequential expression of plant gene maps, regional differentiation characteristics, and intergenerational evolution. The phenotype concept is constantly being redefined. As a result, in the context of the rapid development of phenotyping platforms, the definition of new phenotypic concepts must be determined as soon as possible, and a consistent standard is necessary for correct phenotypic research by scientists.

### Development of a high-performance infrastructure platform

With the widespread adoption of sequencing technology, an increasing number of plant genome sequences have been released, but few functional genes have been identified due to a lack of phenotypic data. Because of leaf occlusion, a substantial amount of information (such as the leaf number, main stem morphology, branch number, branch morphology, fruit number, and fruit morphology) has not been collected during the late growth stage of dicotyledons. The first step in achieving the comprehensive acquisition of high-throughput phenotypic information is to address the loss of original information, which is also the core challenge faced by the high-throughput phenotyping platform. How do you address the issue of lost data? Due to the nature of high-throughput phenotypic information gathering, all of the information that can be extracted is displayed in the original image. Some medical techniques, including computed tomography (CT), magnetic resonance imaging (MRI), and ultrasound, can be used to recover information that has been lost due to leaf occlusion. Unfortunately, because these technologies are both environmentally and financially demanding, they are rarely applied in agriculture.

After more than 10 years of development, crop phenotyping systems have the following characteristics: a high information acquisition efficiency, the use of non-invasive sensors, high-latitude information acquisition, and a resolution that decreases as the information acquisition area expands. As science and technology advance, future high-throughput phenotyping platforms will be able to combine the flux, resolution, dimension, load, robustness, and working height to obtain a large amount of original information efficiently and quickly. Resolution includes both temporal (from seconds to days to months) and spatial (very small, such as for cells, to large, such as for fields and natural environments) dimensions, denoting the variety of phenotypic features acquired by phenotyping systems under various time, space, and scale conditions. The load refers to the maximum weight that a phenotyping platform can carry; notably, in air-based platforms, the load capacity severely restricts the number and variety of sensors that can be used. Therefore, appropriately enhancing the load capacity of a phenotyping platform can promote the diversification of phenotypic data acquisition. The adaptability of phenotyping platforms to work in the field is referred to as robustness. Harsh field conditions pose significant challenges to the normal operation of phenotyping platforms, and enhancing the robustness of a platform can ensure that the platform operates properly. The working height is vital in obtaining phenotypic regions; the obtained regions increase as the working height increases, while the resolution decreases. The key issue for researchers to overcome is how to organically combine the aforementioned factors.

Furthermore, cost control is a critical link, and scientists have been working to determine how to achieve high performance at a low cost. Existing field phenotyping platforms are frequently unable to combine low cost and high performance, failing to meet the needs of most plant phenotype research institutes. As a result, obtaining fast and precise field phenotypic information at a low cost is a bottleneck in the development of a high-throughput phenotyping platform. This necessitates the gathering of talent from various fields, such as machinery, network communication, and sensors, to cross disciplines and contribute to the development of a high-throughput phenotyping platform in the field.

### Construction of a knowledge map of phenotypic big data

Plants are dynamic, complex systems. Plant phenotypes, such as shape, size, color, posture, and texture, will change as they grow. Plants of different varieties have a wide range of appearances at the same time, resulting in the typical 3V characteristics of traditional big data in plant genome big data, that is, data volume, variety, and velocity. A large amount of data is mainly due to the rapid increase in phenotypic data obtained by advanced technology phenotyping equipment based on intelligent equipment and artificial intelligence technology. The diversity and heterogeneity of plant individuals and data types determine data polymorphism. The data are timely because of the dynamic and swift generation of large phenotypic data in the form of data flow.

At the same time, big plant phenomics data exhibit 3H characteristics: high dimension, high complexity, and high uncertainty primarily because plant genomic big data include text data and a large number of images, spectra, and cloud point data, resulting in a wide range of data. The high complexity of phenotypic information is determined by the diversity of genetic information and environmental differences. Phenotypic data have low repeatability and uncertainty because they are affected by many factors, and data acquisition criteria are not uniform. Dicotyledons have more complex plant types and higher 3V and 3H characteristics as a result of genetic diversity and geographical environmental resources, making dicotyledon phenotypic data analysis more difficult. How does one screen for key features under this premise? Big data analysis and in-depth mining must be improved.

After overcoming numerous obstacles to obtain phenotypic data, we are unable to extract such data in depth, resulting in a massive waste of data resources. Phenomic studies are more akin to point-like studies. On the one hand, phenotypic studies are carried out by organizations all over the world, but there is little cooperation between nations and institutions. Currently, only two national-level collaborations (with the US as the sole center and Germany, France, and the United Kingdom as common centers) and four institutional-level collaborations exist (with the United States Department of Agriculture and Cornell University as the centers; China Agricultural University, University of Queensland, Chinese Academy of Sciences, and Chinese Academy of Agricultural Sciences; University of Nottingham and University of Bonn; Wageningen University, French Agricultural Research Institute, and French Scientific Research Center). On the other hand, plant phenotypic databases are limited, and the main data contents vary greatly. For example, the Distributed Phenotypic Data Acquisition and Information Management System (Crop Sight), which primarily includes plant phenotypic data and environmental data (Reynolds et al., 2019a), the phenotypic mixed information system (PHIS), which primarily includes multisource and multiscale information in plant phenomics (Neveu et al., 2019), and the plant genome and phenotypic data sharing platform, primarily consists of data information on plant traits, phenotypes, gene functions, and gene expression of 95 plant taxa (Cooper et al., 2018); the Crop Phenotyping Center of Huazhong Agricultural University, which primarily consists of phenotype data and QTL data (Zhang et al., 2017); and the plant phenotype and genomics data publishing platform. Plant phenotypic data, genome data, mass spectrometry data, and data visualization and analysis software data are mainly included in the PGP Repository (Arend et al., 2016). This phenomenon will result in complex and variable phenotypic data formats, a lack of unified standards, and a significant reduction in the role of data. As a result, we urge countries and institutions to work together to strengthen collaborations, establish phenotype databases, share information, and hold joint discussions.

### Combining with multiomics

The rapid development of high-throughput sequencing, mass spectrometry, and chromatography has facilitated the study of genomics, transcriptomics, proteomics, and metabolomics. Dicotyledons are characterized by complex plant types and a wealth of phenotypic data. When combined with multimers, dicotyledons can unlock more functional genes and facilitate plant genomics research. In breeding practice, high-throughput phenotyping combined with a variety of other omic techniques can be applied to crops in different growth periods and at different scales (cells, tissues, organs, groups) in research on the comprehensive analysis of the calculated crop regulation network of life activity, revealing the biological characteristics of crops. Certain studies on monocotyledons have been conducted in conjunction with multiple omics, such as the study by Leiboff et al. (2015), who used high-throughput image processing technology to determine the size of the shoot apical meristem (SAM) in a natural population of maize and discovered some new candidate genes controlling the SAM size through GWAS analysis. The link between the SAM morphology and trait-related SNP variants was verified after researchers looked into possible genes involved in hormone transport, cell division, and cell size. Xin et al. (2021) employed wild populations of 384 significant wheat varieties (lines) as the basis for a genome-wide association analysis that included phenotypes from three settings and 55K SNP chip typing data. The findings revealed that 142 SNPs were strongly related to the number of spikelets, with phenotypic variance ranging from 3.27 to 6.09%. Using the same strategy, Guo et al. (2018) discovered a novel drought tolerance gene in rice. For dicotyledons, few relevant studies have been conducted: Bac-Molenaar et al. (2015) used the PHENOPSIS phenotype platform to analyze high-throughput images of 324 Arabidopsis cultivars from the top view and, combined with genome-wide association analysis, identified some QTLs related to specific periods and growth rates, revealing a new perspective on the genetic structure of Arabidopsis dynamic development.

The ability to integrate metabolomics approaches into the current HTP phenotypic platform has significant potential to add value (Hall et al., 2022). Metabolites can be divided into volatile and nonvolatile categories, but they all play multiple roles in the plant life cycle. For example, they can be continuously present, having a protective function through antiinsect or antimicrobial activity (Lubes and Goodarzi, 2017; Maurya, 2020). The nonvolatile metabolome represents rich information reflecting past (e.g., slow turnover metabolites accumulated in response to past stress), present (e.g., high turnover metabolic intermediates), and future (e.g., precursors of biomass under construction) events. Accordingly, a growing number of top-down studies have shown that this metabolome can be correlated with performance in panels of genetic diversity (Meyer et al., 2007; Riedelsheimer et al., 2012). Until recently, cost issues have limited metabolomics applications in large-scale phenotyping. However, using high-resolution MS [TOF-MS, Orbitrap, and Fourier transform ion cyclotron resonance mass spectrometry (FT-ICR-MS)] to distinguish different structures with the same nominal mass alongside ultrafast chromatography now makes it possible to combine HTP with a high resolution (Fekete et al., 2014). Systems biology approaches and the use of large numbers of samples have become possible, and increased observations will enable the development of new prebreeding strategies based on predictive models (Fernandez et al., 2021).

We have reason to believe that plant genomics will advance faster with the establishment of relevant research institutions, the improvement of research facilities, the development of software, the convening of international academic conferences, and the formation of relevant academic teams.

The workflow of the high-throughput phenotyping platform is divided into seven steps. The first step is to determine the new phenotypic criteria. The second step is to build a phenotyping platform, including ground-based platforms, air-based platforms, and space-based platforms. The third step is to obtain raw information using visible and invisible sensors. The fourth and fifth steps are the extraction, analysis, and mining of phenotypic information. The next step is to combine phenotypic information with multi-omics. Finally, we aim to solve real problems (including genetic breeding and precision management).

## Author contributions

XL, XX, and MC jointly wrote the article and prepared the figures and tables. MX, WW, CL, LY, and WY provided overall guidance and some references. WL helped to modify and improve the article. All authors contributed to the article and approved the submitted version.

## Funding

This work was supported by the Molecular mechanism of relay intercropping light environment regulating shade-tolerant plant architecture formation in soybean (3217150631), the Physiological mechanism of light regulating branch development in relay intercropping soybean (31871570), the physiology and regulation technology of high quality and high yield of soybean (2018YFD1000905), and the study and demonstration of corn—legume strip compound planting and mixed silage technology in Plateau Tibetan area (2020YFN0021).

## Conflict of interest

The authors declare that the research was conducted in the absence of any commercial or financial relationships that could be construed as a potential conflict of interest.

## Publisher's note

All claims expressed in this article are solely those of the authors and do not necessarily represent those of their affiliated organizations, or those of the publisher, the editors and the reviewers. Any product that may be evaluated in this article, or claim that may be made by its manufacturer, is not guaranteed or endorsed by the publisher.

## Abbreviations

3D: Three Dimensions
CNN: Convolutional Neural Network
CSIRO: Commonwealth Scientific and Industrial Research Organisation
CT: Computed Tomography
DCNN: Deep Convolutional Neural Network
EB: Integrated Baggies
ETH: Ethiopia
εc: Biomass
εe: Photochemical Energy
GWAS: Genome-Wide Association Studies
IPPN: International Plant Phenotype Network
MRI: Magnetic Resonance Imaging
NASA: National Aeronautics and Space Administration
OSP: Optical Sensing-based Phenotyping
PGP: Pretty Good Privacy
PHIS: Phenotypic mixed Information System
QTL: Quantitative Trait Loci
RGB: An abbreviation for three primary colors
SAM: Shoot Apical Meristem
SMAP: Soil Moisture Active Passive
SFM: Multiview Structure From Motion
SNP: Scottish National Party
UAVs: Unmanned Aerial Vehicles
UK: Britain.
